# Legacy effect of fibrate add-on therapy in diabetic patients with dyslipidemia: a secondary analysis of the ACCORDION study

**DOI:** 10.1186/s12933-020-01002-x

**Published:** 2020-03-05

**Authors:** Lin Zhu, Andrew Hayen, Katy J. L. Bell

**Affiliations:** 1 Australian Centre for Public and Population Health Research, Faculty of Health, University of Technology Sydney, Sydney, NSW Australia; 2 School of Public Health, Faculty of Medicine and Health, The University of Sydney, Sydney, NSW Australia

**Keywords:** Fibrate, Dyslipidemia, Type 2 diabetes, Cardiovascular diseases, Legacy effect

## Abstract

**Background:**

The Action to Control Cardiovascular Risk in Diabetes (ACCORD)-Lipid study found no evidence of a beneficial effect of statin-fibrate combined treatment, compared to statins alone, on cardiovascular outcomes and mortality in type 2 diabetes mellitus after 5 years of active treatment. However, a beneficial reduction in major CVD events was shown in a pre-specified sub-group of participants with dyslipidemia. The extended follow-up of this trial provides the opportunity to further investigate possible beneficial effects of fibrates in this group of patients. We aimed to evaluate possible “legacy effects” of fibrate add-on therapy on mortality and major cardiovascular outcomes in patients with dyslipidemia.

**Methods:**

The ACCORD-lipid study was a randomized controlled trial of 5518 participants assigned to receive simvastatin plus fenofibrate vs simvastatin plus placebo. After randomized treatment allocation had finished at the end of the trial, all surviving participants were invited to attend an extended follow-up study (ACCORDION) to continue prospective collection of clinical outcomes. We undertook a secondary analysis of trial and post-trial data in patients who had dyslipidemia. The primary outcome was all-cause and cardiovascular mortality, and secondary outcomes were nonfatal myocardial infarction, stroke, congestive heart failure and major coronary heart disease. We used an intention-to-treat approach to analysis to make comparisons between the original randomized treatment groups.

**Results:**

853 participants with dyslipidemia had survived at the end of the trial. Most participants continued to use statins, but few used fibrates in either group during the post-trial period. The incidence rates in the fenofibrate group were lower with respect to all-cause mortality, CVD mortality, nonfatal myocardial infarction, congestive heart failure and major coronary heart disease than those in the placebo group over a post-trial follow-up. Allocation to the combined fibrate-statin treatment arm during the trial period had a beneficial legacy effect on all-cause mortality (adjusted HR = 0.65, 95% CI 0.45–0.94; *P* = 0.02).

**Conclusions:**

Fibrate treatment during the initial trial period was associated with a legacy benefit of improved survival over a post-trial follow-up. These findings support re-evaluation of fibrates as an add-on strategy to statins in order to reduce cardiovascular risk in diabetic patients with dyslipidemia.

*Trial registration* clinicaltrials.gov, Identifier: NCT00000620

## Background

Dyslipidemia is a major contributor to the increased risk of cardiovascular disease (CVD) among patients with type 2 diabetes mellitus (T2DM). While other types of lipid abnormalities can be found in people with diabetes, the typical diabetic dyslipidemia (also called atherogenic dyslipidemia) is characterized by elevated triglycerides, small dense low-density lipoproteins (LDL) particles, and low levels of high-density lipoproteins (HDL) cholesterol [1]. Recommended first line measures for CVD prevention in people with diabetes who have dyslipidemia include non-drug interventions (dietary regulation, exercise, moderation of alcohol intake and weight loss) and LDL-cholesterol lowering with statin drug therapy [2, 3]. The use of statins as the primary drug treatment option is supported by a large body of evidence. For example, a meta-analysis of 14 randomized trials which included more than 18,000 people with diabetes, found that for every mmol/L reduction in LDL cholesterol there was a 21% proportional reduction in the risk of a major vascular event [4]. This proportional risk reduction is similar to that observed in people without diabetes [5], but because the baseline absolute risk is on average higher in people with diabetes, the absolute benefits are greater. However, the trial data also show substantial “residual risk” in people with T2DM who are on statin treatment [6–8], and often the absolute risk is still higher than that in people without diabetes who are not on statin treatment [9–11]. This indicates that preventative treatment with statins alone may not be enough in people with T2DM and additional therapies may need to be considered. There is also evidence from Mendelian randomization studies that high triglycerides are causally related to CVD, and so drug therapy targeting this lipid abnormality could help to further reduce CVD risk in people with T2DM [12–14].

Fibrates are an example of such a drug therapy, as they both decrease triglyceride levels and increase HDL-C [15]. To investigate if these effects on lipid biomarkers translates into a reduction in CVD, the Action to Control Cardiovascular Risk in Diabetes (ACCORD)-Lipid study randomized 5518 people with T2DM to combined statin-fibrate therapy vs statin therapy alone. Although the ACCORD-Lipid study found no benefit between randomized groups overall, a beneficial reduction in major CVD events was found in a pre-specified sub-group analysis of study participants with dyslipidemia (triglyceride greater than 204 mg/dl and high-density lipoprotein less 34 mg/dl) [16, 17]. The authors hypothesized that fibrate therapy, offered as an add-on to statin therapy, may be beneficial for people with diabetes who are found have hypertriglyceridemia and/or reduced HDL-C. This hypothesis is supported by the findings of several systematic reviews of RCTs of fibrate therapy [18–21].

At the end of the ACCORD-Lipid trial, participants were unblinded from their randomized groups, and passively followed up for an additional 5 years through follow-up clinics and routine data collection methods. The post-trial follow-up data provide a unique opportunity to evaluate the effect of add-on fibrate therapy in the longer-term, and the possibility of the emergence of “legacy effects”. Legacy effects describe intervention effects observed in the post-trial period which are not due to the direct effects observed during the trial period [22]. The finding of a legacy effect would have important clinical implications, including the potential benefits of early initiation of fibrate treatment in the setting of diabetic dyslipidemia. Although potential legacy effects for statin treatment have been investigated in a number of post-trial follow up studies [23], those for combined statin-fibrate treatment remain unexplored [24, 25]. Post-trial data after a statin-fibrate RCT provide the opportunity to investigate potential legacy effects in people with T2DM and dyslipidemia. Therefore, we conducted a secondary analysis of data from the ACCORD-Lipid trial and the ACCORDION post-trial follow-up study, in order to determine whether or not there is evidence for legacy effects for fibrate add-on strategy to statins in diabetic patients with dyslipidemia.

## Methods

### Study participants and setting

The Action to Control Cardiovascular Risk in Diabetes (ACCORD) Trial was a randomized, double 2 × 2 factorial design study, which evaluated the effects of intensive glycemic control, intensive blood pressure control, and combined fibrate statin treatment, on the prevention of cardiovascular disease in people with T2DM [26]. It enrolled 10,251 people (mean age 62 years), who had a history of T2DM for a median duration of 10 years, with mean glycated hemoglobin (HbA1c) level of 8.3%. Participants had either a history of previous cardiovascular disease or had elevated risk factors levels. The lipid sub-study was conducted in 5518 of the trial participants. In addition to fulfilling the overarching ACCORD entry criteria, the LIPID participants needed to meet all of the following additional criteria: (1) 60 mg/dl < LDL-C < 180 mg/dl (1.55 to 4.65 mmol/l) if not on a lipid lowering agent during screening, or, if on a lipid-lowering agent, the LDL-C needed to be between prespecified drug/dose-specific cut points, and (2) HDL-C less than 55 mg/dl (1.42 mmol/l) for women or African-Americans, or HDL-C less than 50 mg/dl (1.29 mmol/l) for all other gender and ethnic groups, and (3) triglycerides < 750 mg/dl (8.47 mmol/l) on no therapy or < 400 mg/dl (4.52 mmol/l) on treatment with lipid lowering drugs. Participants were randomly assigned to either simvastatin plus fenofibrate or simvastatin plus placebo. The starting dose of open-labeled simvastatin were determined by presence of cardiovascular disease and the dose of masked fenofibrate/placebo were determined by calculated glomerular filtration rate at randomization. Further changes to the dose of both drugs were made during the trial in accordance to the trial guidelines [16]. At the end of the trial, all surviving ACCORD participants who could be contacted were invited to enter an observational follow-up study (ACCORDION) [27, 28]. No active trial therapy was provided in this period, and medical care was provided by the participant’s local primary care provider. Data on health outcomes (e.g. hospital records, death certificates, etc.) and medication usage were collected by phone and clinic visits. Physical examinations were conducted at the first and last clinic visits, include the collection of urine and blood samples for analysis [28].

In the ACCORD-Lipid trial, dyslipidemia was pre-specified as the combination of the highest tertile of triglyceride (204 mg/dl) and lowest third of HDL-C (34 mg/dl) at baseline [16]. We used the same definition for dyslipidemia in the current analysis. Our primary outcomes were all-cause mortality and cardiovascular mortality, and our secondary outcomes were nonfatal myocardial infarction, stroke, congestive heart failure and a major coronary heart disease event [16, 28]. Although there was event adjudication during the ACCORD trial, this was done in only a randomly selected 10% of events during the post-trial follow-up period (for the purpose of quality control). For consistency across all follow up data, we used outcomes reported by site investigators during both trial and post-trial period (unadjudicated events).

### Statistical methods

Participants’ characteristics at baseline of trial and first post-trial visit were summarized for the two randomized groups using means, standard deviations, and percentages. The measured lipid levels at each study visit, including total cholesterol, triglycerides, HDL-C, LDL-C and VLDL-C, were compared between randomized groups. VLDL-C was obtained by subtracting HDL-C and LDL-C from total cholesterol. Primary and secondary outcomes were analyzed according to the intention-to-treat principle. Hazard ratios (HRs) and 95% confidential intervals were estimated using Cox proportional hazards models. Kaplan–Meier estimates were used to obtain the proportion of patients who had an event during follow-up. The direct effects of treatment were estimated by fitting models for the trial period (short term effects), and the entire study period (from baseline of trial through to end of post-trial, long term effects). The legacy effects of treatment were estimated by fitting models for the post-trial period alone. This analysis was based on survivors who consented to additional follow-up, and their follow-up times were calculated by the difference between full follow-up time and censoring time for the trial. These analyses were adjusted for age, sex, ethnicity, network, education status, CVD history, blood glucose trial treatment assignment and years of diabetes. To examine the robustness of these findings, sensitivity analyses were undertaken to (i) account for effects of medications taken in the post-trial follow-up period, and (ii) to account for possible imbalance in confounders between the two groups at the start of post-trial follow-up (using inverse probability weighting). All analyses were performed with R (version 3.5.1).

## Results

### Characteristics of the participants at baseline and 1st post-trial visit

Of a total of 5518 patients enrolled in the ACCORD Lipid trial, 940 (17.0%) were identified as having dyslipidemia. 484 of them were assigned to fenofibrate and simvastatin therapy, and 456 participants received simvastatin and placebo. Of these participants, 853 had survived at the end of the trial, and 765 (90.0%) consented to enter the post-trial follow-up study. The median follow-up time in the post-trial period was 4.9 years. Table 1 shows characteristics of the participants at the trial baseline and at the first post-trial visit. The mean age at baseline was 61.8 years, and the fenofibrate group was slightly younger than the placebo group. Most of the patients were male and about forty percent of patients had a history of CVD disease. The HbA1c, blood pressure and lipid levels were well matched across treatment groups both at baseline and 1^st^ post-trial visit.

**Table 1:** Characteristics of the participants at baseline and 1st post-trial visit

Characteristics	Baseline		P	1st post-trial visit		P
	Fenofibrate (n = 484)	Placebo (n = 456)		Fenofibrate (n = 395)	Placebo (n = 370)	
Age	61.4 ± 6.2	62.2 ± 6.7	0.04	67.2 ± 6.3	67.6 ± 6.6	0.32
Sex			0.96			0.59
Male	388 (80.2%)	364 (79.8%)		319 (80.8%)	292 (78.9%)	
Female	96 (19.8%)	92 (20.2%)		76 (19.2%)	78 (21.1%)	
Years of diabetes	9.2 ± 6.6	9. 6 ± 6.6	0.37	14.6 ± 6.5	15.2 ± 6.6	0.22
Ethnicity			0.17			0.51
White	365 (75.4%)	362 (79.4%)		305 (77.2%)	294 (79.5%)	
Non-White	119 (24.6%)	94 (20.6%)		90 (22. 8%)	76 (20.5%)	
CVD history			0.93			0.98
Yes	195 (40.3%)	186 (40.8%)		153 (38.7%)	142 (38.4%)	
No	289 (59.7%)	270 (59.2%)		242 (61.3%)	228 (61.6%)	
BG trial assignment			0.36			0.36
Intensive group	251 (51.8%)	222 (48.7%)		203 (51.4%)	177 (47.8%)	
Standard group	233 (48.2%)	234 (51.3%)		192 (48.6%)	193 (52.2%)	
HbA1c (%)	8.4 ± 1.1	8.4 ± 1.0	0.94	7.9 ± 1.9	7.6 ± 1.3	0.07
SBP (mm Hg)	134.1 ± 17.6	133.9 ± 18.6	0.87	131.0 ± 17.1	131.8 ± 17.3	0.66
CHOL (mg/dl)	187.0 ± 38.5	189.0 ± 42.1	0.45	154.6 ± 42.5	152.8 ± 32.8	0.64
TG (mg/dl)	327.2 ± 125.3	325.0 ± 154.2	0.81	216.6 ± 124.1	222.7 ± 115.2	0.61
VLDL-C (mg/dl)	61.2 ± 18.6	61.2 ± 25.4	0.97	41.4 ± 20.8	42.63 ± 20.1	0.55
LDL-C (mg/dl)	96.3 ± 32.0	98.4 ± 32.9	0.34	79.2 ± 32.7	76.6 ± 26.1	0.38
HDL-C (mg/dl)	29.5 ± 3.8	29.5 ± 3.7	0.76	33.9 ± 7.3	33.6 ± 7.2	0.60

Plus–minus values are mean ± SD

*HbA1c* glycated hemoglobin A1c, *SBP* systolic blood pressure, *CHOL* total cholesterol, *TG* triglyceride, *HDL-C* high-density lipoprotein cholesterol, *LDL-C* low-density lipoprotein cholesterol, *VLDL-C* very low-density lipoprotein cholesterol

### Trial adherence and use of lipid-modifying medication after trial

Participants’ adherence during the trial and the use of statin/fibrate post-trial is shown in Table 2. The adherence for both simvastatin and fenofibrate/placebo during the trial period was high. In the post-trial period, most participants continued to use statin therapy, while few used fibrates in either group (likely due to the finding of no benefit overall in the ACCORD-Lipid study).

**Table 2:** Trial adherence and use of lipid-modifying medication post-trial

Time	Treatment	Proportion on-treatment (%)	
		Fenofibrate group	Placebo group
Year 1	Fenofibrate/placebo	91.4	91.2
	Simvastatin	94.5	95.5
Year 2	Fenofibrate/placebo	88.8	91.5
	Simvastatin	93.6	96.1
Year 3	Fenofibrate/placebo	87.2	90.2
	Simvastatin	91.3	92.9
Year 4	Fenofibrate/placebo	85.3	86.3
	Simvastatin	92.9	92.5
Trial exit visit	Fenofibrate/placebo	82.7	86.3
	Simvastatin	93.1	91.0
1st post-trial visit	Fibrate	7.2	7.4
	Stains	77.1	78.4
Last post-trial visit	Fibrate	5.4	4.8
	Stains	72.0	74.0

### Efficacy of fenofibrate in lipid-modifying

**Fig. 1:**
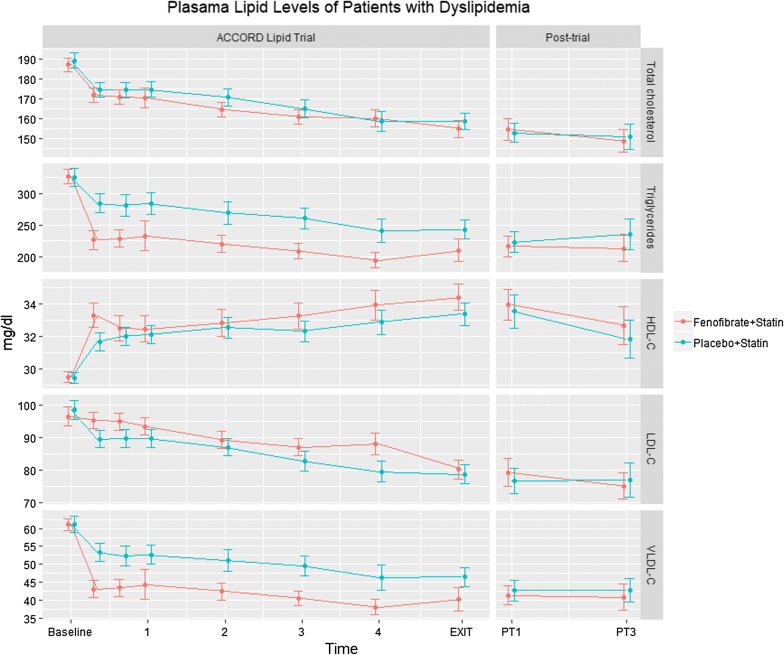
Plasma lipid levels of patients with dyslipidemia at each study visit. The line charts show the means of lipid levels and corresponding 95% CI at 1/2/3/4 year, exit visit, 1st post-trial visit and last post-trial visit. *HDL-C* high-density lipoprotein cholesterol, *LDL-C* low-density lipoprotein cholesterol, *VLDL-C* very low-density lipoprotein cholesterol, *PT1* first post-trial clinic visit, *PT3* last post-trial clinic visit

Figure 1 compares the plasma lipids of the two groups at each study visit during the within trial and post-trial periods. During the trial, allocation to fenofibrate resulted in improvements in almost all lipids compared with placebo, but the largest differences were seen for plasma triglyceride concentrations and VLDL-C levels. Further, although the differences in HDL-C and LDL-C levels between randomized groups decreased over time, they were maintained for levels of triglycerides (P = 0.01) and VLDL-C (P = 0.006) through to the end of the trial. At the first post-trial visit there were minimal differences between randomized groups for any of the lipids, and this remained the case through to the last clinic visit.

### Clinical outcomes

**Fig. 2:**
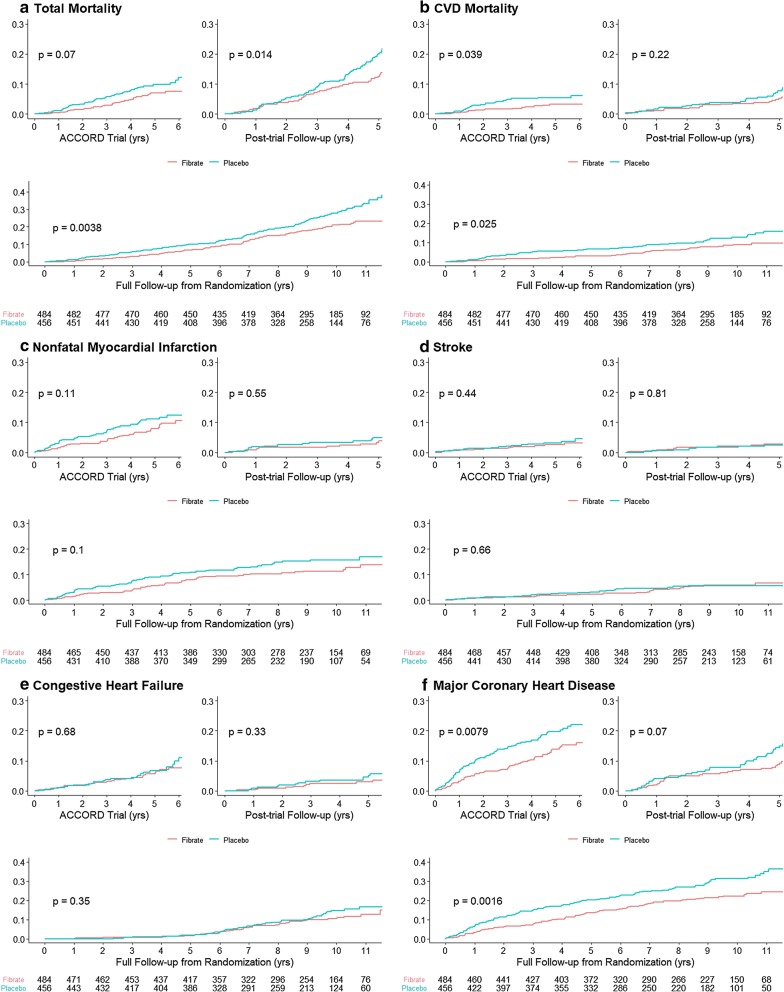
Kaplan–Meier cumulative event curves for primary and secondary outcomes. The Kaplan–Meier curves display the time to event for the all-cause mortality (**a**) and cardiovascular mortality (**b**), nonfatal myocardial infarction (**c**), stroke (**d**), congestive heart failure (**e**) and a major coronary heart disease event (**f**) during trial period, post-trial and the entire study period. The numbers of individuals at risk are shown for each time point

The incidence rate of the primary and secondary outcomes and the hazard ratios of allocation to the fenofibrate plus simvastatin versus simvastatin alone during the ACCORD-lipid trial, ACCORDION and the full follow-up period are shown in Table 3. We found that the incidence rates in the fenofibrate group were lower with respect to all-cause mortality, CVD mortality, nonfatal myocardial infarction, congestive heart failure and major coronary heart disease than those in the placebo group over the post-trial follow-up. Allocation to the combined fibrate-statin treatment arm during the trial period resulted in a statistically significant beneficial legacy effect on all-cause mortality observed in the post-trial period (adjusted HR = 0.65, 95% CI 0.45–0.94; P = 0.02, other effects not statistically significant). Long-term beneficial effects were also found when trial and follow up periods were combined (9.7 years follow-up from time of randomization) for all-cause mortality, CVD mortality and major coronary heart disease events (effects on CVD mortality and all-cause mortality were statistically significant). Kaplan–Meier cumulative event curves for primary outcome and selected secondary outcomes are consistent with findings from the Cox models and are presented in Fig. 2. Sensitivity analyses adjusting for medication use of post-trial follow-up and for other potential confounders, using inverse probability weighting, resulted in similar findings (Additional file 1: Table S1).

**Table 3:** Clinical outcomes by randomized treatment during ACCORD-lipid trial, ACCORDION and full follow-up period

Event	During ACCORD-lipid (short-term effect)			*P*	Post-trial only (legacy effect)			*P*	Full follow-up (long-term effect)			*P*
	Rate of events (100 person-years)		Hazard Ratio (95% CI)		Rate of events (100 person-years)		Hazard ratio (95% CI)		Rate of events (100 person-years)		Hazard ratio (95% CI)	
	Fibrate	Placebo			Fibrate	Placebo			Fibrate	Placebo		
All-cause mortality	1.54	2.26	0.68 (0.44, 1.04)	0.07	3.05	4.43	0.65 (0.45, 0.94)	0.02	2.23	3.24	0.68 (0.52, 0.88)	< 0.01
CVD mortality	0.67	1.28	0.53 (0.29, 0.98)	0.04	1.12	1.53	0.77 (0.43, 1.39)	0.38	0.88	1.39	0.63 (0.42, 0.95)	0.03
Nonfatal MI	1.76	2.45	0.72 (0.47, 1.09)	0.12	0.85	1.07	0.74 (0.33, 1.66)	0.47	1.40	1.91	0.74 (0.51, 1.06)	0.10
Stroke	0.56	0.74	0.75 (0.36, 1.56)	0.44	0.56	0.55	0.87 (0.33, 2.29)	0.78	0.58	0.67	0.88 (0.5, 1.56)	0.66
CHF	1.39	1.51	0.90 (0.55, 1.47)	0.68	0.65	0.93	0.69 (0.30, 1.57)	0.38	1.07	1.27	0.82 (0.54, 1.24)	0.35
Major CHD	3.01	4.60	0.65 (0.48, 0.90)	0.01	2.17	3.27	0.64 (0.40, 1.03)	0.07	2.67	4.09	0.66 (0.51, 0.86)	< 0.01

*MI* myocardial infarction, *CHF* congestive heart failure, *CHD* coronary heart disease

## Discussion

We found that patients with dyslipidemia who were randomized to statin-fibrate treatment during the trial had higher survival in the 5 years after the trial than those randomized to statin-placebo. This effect was observed despite similar achieved lipid profile during the extended observational follow-up, which suggests a legacy effect of fibrate add-on therapy on all-cause mortality. Although estimated legacy effects on all other outcomes were not statistically significant, the effect estimates suggest that improved survival is likely to be largely explained through effects on CVD. No information was available on non-CVD causes of death, which meant we were not able to explore other possible explanations for the all-cause mortality reduction. The overall long-term benefits for CVD mortality appeared to be driven by both within-trial treatment effects and legacy effects emerging post-trial. Other studies suggest that fibrates may also have beneficial effects on microvascular outcomes, including on renal and liver function [29], but we didn’t have data to explore this.

During the trial period, fibrate add-on therapy reduced triglycerides and VLDL-C beyond that achieved with statins only, but HDL-C was increased by only a limited amount. These findings have been observed in other clinical trials of fibrate—for example in the Fenofibrate Intervention and Event Lowering in Diabetes (FIELD) study, allocation to fenofibrate resulted in a 20% reduction of baseline TG, but HDL-C remained almost unchanged at study close [30–32]. The improvement of the triglyceride-rich environment may explain the reduced risk of CVD observed during the trial period in these patients [15, 33]. As most of participants in active arm discontinued the use of fibrate in post-trial, between group differences in triglycerides and VLDL-C soon disappeared. This suggests continuous treatment is necessary to maintenance a lower TRIG/VLDL-C.

Our findings on potential beneficial effects on CVD mortality reduction are supported by a recent report of a large propensity matched cohort study that found a (non-statistically significant) reduction in CVD mortality associated with fibrate use [23]. Results from the ongoing Pemafibrate to Reduce Cardiovascular Outcomes by Reducing Triglycerides in Patients with Diabetes (PROMINENT) study will also provide evidence regarding short term effectiveness; further follow up studies are needed for longer term legacy effects [34, 35].

Our study has several limitations. First, our analysis examined a relatively small subset of the full trial and the power to detect smaller effects is limited [36]. The findings for this prespecified subgroup with dyslipidemia must be interpreted with caution, and further larger studies in people with dyslipidemia are needed. Second, as in ACCORD, the diabetic dyslipidemia was defined in a data-driven manner, however the thresholds used are similar to other definitions of dyslipidemia [3, 37]. Third, we used the investigators reported (unadjudicated) cause of death data for both trial and post-trial periods. A previous report from ACCORD study group has shown the CVD mortality was under-reported by the investigators compared to the adjudicated Committee [28], suggesting potential misclassification of cause of death using these data. Fourth, although we adjusted analyses for potential imbalance between randomized groups in confounders during the post-trial period, measurement error in these, and the presence of other unmeasured confounders could bias our estimates [22].

## Conclusion

In conclusion, this secondary analysis found evidence of legacy effects of fenofibrate-statin combined therapy on all-cause mortality in diabetic patients with dyslipidemia. This finding suggests fibrate treatment may be an effective means of reducing residual cardiovascular risk in these patients.

## Supplementary information

**Additional file 1.** Results of the sensitivity analysis.

## Data Availability

The datasets analyzed during the current study are available from the National Heart, Lung, and Blood Institute on reasonable request.

## Abbreviations

CVD: Cardiovascular disease
T2DM: Type 2 diabetes mellitus
ACCORD: Action to Control Cardiovascular Risk in Diabetes
ACCORDION: Action to Control Cardiovascular Risk in Diabetes Follow-On
TRIG: Triglyceride
HDL: High-density lipoprotein
LDL: Low-density lipoprotein
VLDL: Very low-density lipoprotein
MI: Myocardial infarction
CHF: Congestive heart failure
CHD: Major coronary heart disease
