# Supplementary material for: Legacy effect of fibrate add-on therapy in diabetic patients with dyslipidemia: a secondary analysis of the ACCORDION study
Source: Cardiovasc Diabetol. 2020 Mar 5;19:28. doi: 10.1186/s12933-020-01002-x (PMC7059389; doi:10.1186/s12933-020-01002-x)
Supplement: Supplementary file 1 — Additional file 1. Results of the sensitivity analysis. [file 12933_2020_1002_MOESM1_ESM.docx]

**Sensitivity Analysis**

To examine the robustness of our findings, we undertook a sensitivity analysis of legacy effect by modelling in the following ways:

Model 1. Unadjusted Model

Model 2. Adjusted for baseline Characters (age, gender, race, education, CVD history, BG trial treatment assignment, duration of T2MD) and post-trial medication use (medication of blood glucose control, blood pressure control, statin, and fibrate)

Model 3. Inverse probability weighting (IPW). Individuals were reweighted based on their probability of survival for each specific event. The probability was estimated using baseline age, gender, ethnicity, education, CVD history, clinical center, smoking status, alcohol, baseline medication use, BG trial treatment assignment, baseline HbA1c, systolic blood pressure, TRIG, LDL-C and HDL-C.

**Table S1.** Result of sensitivity analysis

| Event | Model 1 | | Model 2 | | Model 3 | |
| --- | --- | --- | --- | --- | --- | --- |
|  | Hazard Ratio (95%CI) | P | Hazard Ratio (95%CI) | P | Hazard Ratio (95%CI) | P |
| Total mortality | 0.64(0.45, 0.91) | 0.01 | 0.54(0.30, 0.99) | 0.05 | 0.57(0.35, 0.94) | 0.03 |
| CVD mortality | 0.78(0.44, 1.38) | 0.39 | 0.59(0.24, 1.47) | 0.26 | 0.82(0.43, 1.55) | 0.55 |
| Nonfatal MI | 0.85(0.39, 1.84) | 0.68 | 0.52(0.19, 1.45) | 0.21 | 1.00(0.44, 2.30) | 0.99 |
| Total stroke | 1.00(0.39, 2.6) | 0.99 | 1.33(0.42, 4.21) | 0.63 | 1.16(0.43, 3.16) | 0.77 |
| CHF | 0.68(0.31, 1.49) | 0.33 | 0.46(0.17, 1.24) | 0.12 | 0.75(0.33, 1.69) | 0.50 |
| Major CHD | 0.65(0.41, 1.04) | 0.07 | 0.47(0.24, 0.92) | 0.03 | 0.50(0.27, 0.89) | 0.02 |
